# Decreased expression of B cell related genes in leukocytes of women with Parkinson's disease

**DOI:** 10.1186/1750-1326-6-66

**Published:** 2011-09-23

**Authors:** Merav Kedmi, Anat Bar-Shira, Tanya Gurevich, Nir Giladi, Avi Orr-Urtreger

**Affiliations:** 1 Genetic Institute, Tel Aviv Sourasky Medical Center, 6 Weizmann Street, Tel Aviv 64239, Israel; 2 Movement Disorders Unit and Parkinson Center, Department of Neurology, Tel Aviv Sourasky Medical Center, 6 Weizmann Street, Tel Aviv 64239, Israel; 3 The Sackler Faculty of Medicine, Tel Aviv University, Tel Aviv 69978, Israel

## Abstract

**Background:**

Parkinson's disease (PD) is a complex disorder caused by genetic, environmental and age-related factors, and it is more prevalent in men. We aimed to identify differentially expressed genes in peripheral blood leukocytes (PBLs) that might be involved in PD pathogenesis. Transcriptomes of 30 female PD-patients and 29 age- and sex-matched controls were profiled using GeneChip Human Exon 1.0 ST Arrays. Samples were from unrelated Ashkenazi individuals, non-carriers of *LRRK2 *G2019S or *GBA *founder mutations.

**Results:**

Differential expression was detected in 115 genes (206 exons), with over-representation of immune response annotations. Thirty genes were related to B cell functions, including the uniquely B cell-expressed *IGHM *and *IGHD*, the B cell surface molecules *CD19*, *CD22 *and *CD79A*, and the B cell gene regulator, *PAX5*. Quantitative-RT-PCR confirmation of these 6 genes in 79 individuals demonstrated decreased expression, mainly in women patients, independent of PD-pharmacotherapy status.

**Conclusions:**

Our results suggest that the down regulation of genes related to B cell activity reflect the involvement of these cells in PD in Ashkenazi individuals and represents a molecular aspect of gender-specificity in PD.

## Background

Parkinson's disease (PD) is a complex disorder involving multiple affected genes and multiple environmental risk factors [1]. It is the second most common neurodegenerative disorder, affecting about 1.8% of the population over the age of 65 years [2]. Since the identification of mutations in the α-synuclein gene [3], 16 chromosomal loci, and mutations in 9 genes have been associated with familial and sporadic PD (reviewed by [4]). Abnormalities in multiple cellular pathways including the ubiquitin-proteasome, mitochondrial and apoptotic pathways and impaired protection from oxidative stress were suggested to be involved in the accumulation of synuclein and the selective loss of dopaminergic and other neurons (reviewed by [5]). Transcriptional profiling by microarray methodology of the substantia nigra (SN) from PD-patients [6-8], as well as from peripheral blood leukocytes (PBL) from PD-patients [9,10] further demonstrated expression changes in genes belonging to these pathways. The identification of similar expression changes in SN and PBL encouraged our use of PBL as a surrogate tissue to search for novel genes and cellular pathways that might be involved in PD pathogenesis.

Genetic analysis of isolated populations derived from relatively few founders offers a powerful route for the identification of genetic risk factors [11]. The Ashkenazi Jewish population has preserved its homogeneous genetic makeup, and has been valuable for the identification of genes associated with increased risk for many common complex diseases, relevant to the world population-at-large [12,13]. In our previous studies, mutations in the *LRRK2 *and *GBA *genes were detected in a surprisingly high proportion (more than a third) of the Ashkenazi PD-patients tested [14,15], allowing the sub-classification of these patients based on their *LRRK2 *or *GBA *carrier status.

Gender differences in PD are well characterized, with PD prevalence being about 1.5 times lower in women than in men in Western populations [16,17]. Phenotypic changes between men and women have also been described, for example, age at PD onset is ~3 years later in women [16,17], and men with PD have higher risk of developing cognitive impairment [18].

To increase the possibility of identifying PD-related expression changes in patients' PBL, we studied the relatively genetically homogenous population of Ashkenazi patients and controls. To further increase the homogeneity of the studied population in the initial step of the microarray expression profiling, we included only female patients and controls that do not carry the *LRRK2 *or *GBA *Ashkenazi founder mutations. The microarray methodology was designed to capture the expression intensity of each exon individually, allowing the identification of differentially expressed genes in PD-patients compared to healthy controls.

## Results

### Quality control and batch effects removal

Principal component analysis (PCA) revealed two methodological factors that affected the expression levels, the Affymetrix whole-transcript target labeling kit batches (2 batches, Additional file 1, Figure S1, A) and the two researchers who extracted the RNAs (Additional file 1, Figure S1, B). Successful elimination of the batch effects and data homogeneity were demonstrated in PCA done following the batch-removed ANOVA function (Partek^® ^Genomics Suite Version 6.4, Additional file 1, Figures S1, C and D).

### Expression changes detected in PBL from Parkinson's disease patients

**Figure 1:**
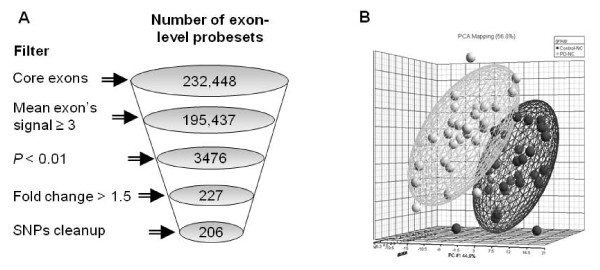
**Significant expression changes in peripheral blood leukocytes of Parkinson's disease patients compared to controls**. (**A**) Schematic representation of the statistical analysis and the filters applied on the Affymetrix GeneChip Human Exon 1.0 ST Array exon-level expression data. The numbers of exon-level probesets remained after each filtering step are indicated in circles. (**B**) PCA mapping of patients and control samples according to the expression levels of the 206 significantly changed exon probesets.

Of the 232,448 core exon-level probesets data in the Human Exon 1.0 array, 195,437 had mean signal value of 3.0 or more in all samples, 227 of them were significantly changed between PD-patients and controls (3-way ANOVA, *P *< 0.01 and fold-change (FC) > 1.5 or < -1.5). Filtering out probesets with known SNPs resulted in 206 probesets that were incorporated into 115 genes (Figure 1A and 1B; Additional file 2, Table S1). 160 probesets (75 genes) were down-regulated and 46 probesets (40 genes) were up-regulated in PD-patients' PBL compared to controls. Alternative-splicing, including possible exon skipping and alternative 3' or 5' sites, was detected in 76 out of the 115 differentially expressed genes using the Partek's gene view tool (Additional file 2, Table S1). Interestingly, 13 of the 115 differentially expressed genes have previously been reported to be changed in the substantia nigra (SN) from PD-patients [6,8,19-22]. In five of these genes (*ADCY2*, *CCDC92*, *CELSR1*, *HECTD2 *and *KIF1B*) the direction of expression change in patients' PBL were as those reported in dopaminergic neurons captured by laser microdissection from patients' SN [6,19]. Twelve of the differentially expressed genes (10.4%) likely belong to PD-related pathways based on their GO annotations (DAVID functional analysis tools, http://david.abcc.ncifcrf.gov/): the ubiquitin-proteasome pathway (*FBXL13*, *HECTD2 *and *PSMC5*), mitochondrial dysfunction (*ACSM5*, *KIF1B*, and *MMAA*); increased oxidative stress (*GPX3*) and apoptosis and cell death (*CKAP2*, *DDIT4*, *IFNG*, *NEB *and *TRAJ17*).

### Genes associated with immune response, specifically with B cell-related functions, are differentially expressed in PD-patients

**Figure 2:**
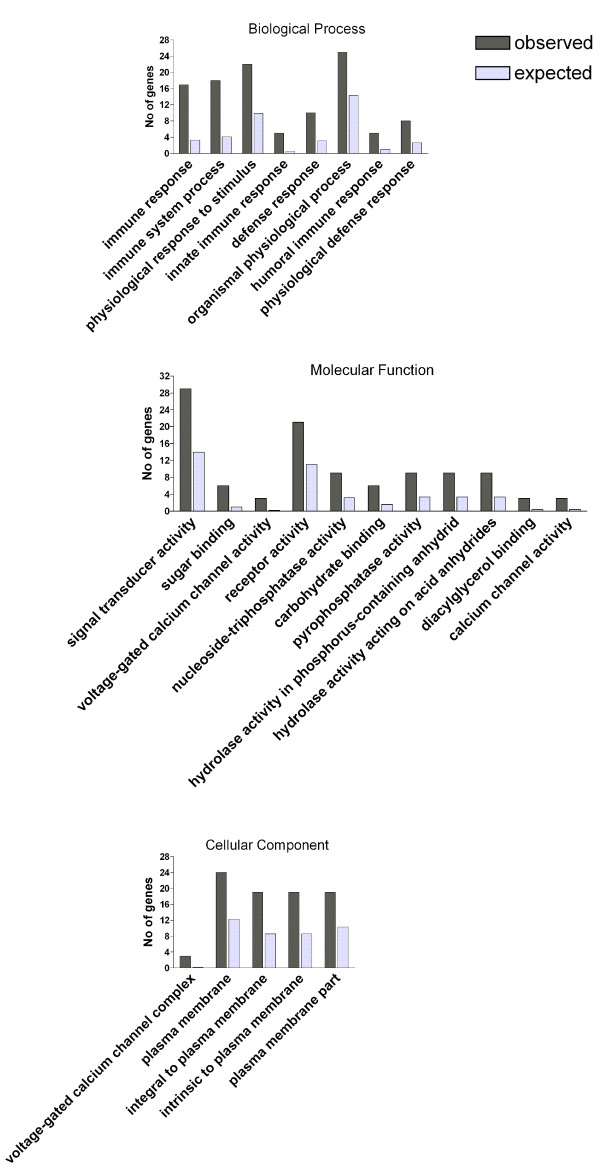
**Functional annotation analysis**. Significantly over-represented GO annotations were detected among the 115 changed genes (206 exon probesets). Only GO annotations with at least 3 genes and *P *< 0.01 (Fisher exact probability) are presented here. The complete data of enriched GO annotations with *P *< 0.05 and a threshold of 2 genes are available in Additional file 3, Table S2. For each annotation, the dark bar represents the number of observed genes among the differentially expressed genes, and the grey bar represents the number of expected genes among the core-level Human Exon 1.0 ST Array.

Significantly enriched GO-annotations were identified when comparing their representation in the 115 significantly changed genes to their representation on the Human Exon 1.0 ST array (21,980 genes, Figure 2 and Additional file 3, Table S2). Genes involved in the immune response were most significantly enriched (17 genes, *P *= 2.69*10^-8^), including genes involved in the innate and humeral immune responses (5 genes each, *P *= 2.29*10^-4 ^and, *P *= 3.85*10^-3 ^respectively). Additional enriched functional annotations were genes with signal transduction activity (29 genes [*P *= 6.17*10^-5^], 21 of them with receptor activity [*P *= 2.6*10^-3^], mostly immuno-receptors) and voltage-gated ion channels (5 genes [*P *= 1.13*10^-2^], 3 of them calcium channels [*P *= 1.11*10^-3^]). Of note, the same significantly enriched annotations were also identified when we compared the representations of the GO-annotations between the 115 differentially expressed genes and the list of genes expressed in our PBL samples (17,441 genes, data not shown).

Significantly over-represented KEGG pathways were also identified (Table 1), and the most significantly enriched pathway was "B cell receptor signaling" (8/115 genes, *P *= 2.21*10^-8^, 19.1 times higher than expected; Table 2A). Notably, such enrichment was not detected in other lymphocyte signaling pathways, such as T cell receptor signaling and Natural killer cell mediated cytotoxicity, but was unique to the B cell pathway.

**Table 1:** Enriched KEGG pathways among the 115 changed transcripts

KEGG pathway	Genes in pathway	Enrichment
B cell receptor signaling pathway	*BLNK, CD19, CD22, CD72, CD79A, CD79B, CR2, RASGRP3*	O = 8;E = 0.419;R = 19.111;P = 2.21e^-8^
Hematopoietic cell lineage	*CD19, CD22, CD8B, CR2, FCER2, IL1R2*	O = 6;E = 0.546;R = 10.995;P = 2.60e^-5^
Type II diabetes mellitus	*CACNA1C, CACNA1D, IRS2*	O = 3;E = 0.284;R = 10.560;P = 3.45e^-3^
Calcium signaling pathway	*ADCY2, CACNA1C, CACNA1D, GNAL, P2RX5*	O = 5;E = 1.114;R = 4.489;P = 5.78e^-3^
MAPK signaling pathway	*CACNA1C, CACNA1D, CACNB4, IL1R2, RASGRF1, RASGRP3*	O = 6;E = 1.779;R = 3.373;P = 9.64e^-3^
ABC transporters - General	*ABCA13, ABCB4*	O = 2;E = 0.254;R = 7.868;P = 2.92e^-2^
GnRH signaling pathway	*ADCY2, CACNA1C, CACNA1D*	O = 3;E = 0.650;R = 4.613;P = 2.93e^-2^
Cell adhesion molecules (CAMs)	*CD22, CD40, CD8B*	O = 3;E = 0.807;R = 3.716;P = 4.95e^-2^

O, Observed number of genes carrying the annotation among the 115 differentially expressed genes; E, expected number of genes that carry this annotation among all core-level genes on the array; R, ratio between observed and expected; P, *P*-value of the enrichment, calculated using Fisher exact test.

**Table 2:** B cell-related genes with significantly changed expression in PBL of PD-patients

Symbol	GenBank Accession No	Gene Name	B cells involvement
**A. *Genes involved in B cell receptor signaling pathway ***			

BLNK	NM_013314	B-cell linker	B cell receptor signaling pathway (a); B cell activation (b), (g); B cell differentiation (b)
CD19	NM_001770	CD19 antigen	B cell receptor signaling pathway (a); Expressed in B-cell (f), (g)
CD22	NM_001771	CD22 antigen	B cell receptor signaling pathway (a); Expressed in B-cell (f), (g)
CD72	NM_001782	CD72 antigen	B cell receptor signaling pathway (a); Expressed in B-cell (g)
CD79A	NM_001783	CD79A antigen (immunoglobulin-associated alpha)	B cell receptor signaling pathway (a),(b); B cell differentiation (b); B cell proliferation (b); B cell receptor complex (c)
CD79B	NM_000626	CD79B antigen (immunoglobulin-associated beta)	B cell receptor signaling pathway (a),(b); B cell receptor complex (c)
CR2	NM_001006658	complement component (3d/epstein barr virus) receptor 2	B cell receptor signaling pathway (a); B Lymphocyte Cell Surface Molecules (d)
RASGRP3	NM_170672	ras guanyl releasing protein 3 (calcium and dag-regulated)	B cell receptor signaling pathway (a)

**B. *Genes involved in other B cell-related annotations***			

BANK1	NM_017935	B-cell scaffold protein with ankyrin repeats 1	B cell activation (b)
CD40	NM_001250	CD40 antigen (tnf receptor superfamily member 5)	Positive regulation of B cell activation (b); Positive regulation of B cell proliferation (b); B cell mediated immunity (b)
EBF1	NM_024007	early b-cell factor	B Cell Development (e)
FCER2	NM_002002	FC fragment of ige, low affinity ii, receptor for (cd23a)	Expressed in B-cell (f), (g)
IFNG	NM_000619	interferon, gamma	Positive regulation of B cell activation (b); B cell mediated immunity (b)
IGHD	BC021276	immunoglobulin heavy constant delta	Expressed in primary B-Cells (f)
IGHM	BC001872	immunoglobulin heavy constant mu	Positive regulation of B cell proliferation B cell receptor signaling pathway (b); B cell receptor complex (c); B Cell Development; B cell activation (e); Expressed in primary B-Cells (f)
IL1R2	NM_004633	interleukin 1 receptor, type ii	Expressed in B-cell (f)
MSC	NM_005098	musculin (activated b-cell factor-1)	Expressed in B-cell (f)
SPIB	NM_003121	spi-b transcription factor (spi-1/pu.1 related)	Expressed in B-cell (f)
TCL1A	NM_021966	T-cell leukemia/lymphoma 1a	Expressed in B-cell (f)

**C. *Additional B cell-related genes***			

CD180	NM_005582	CD180 antigen	Expressed mainly on mature B cells (h); May be involved in the life/death decision of B-cells (j)
CRISP3	NM_006061	cysteine-rich secretory protein 3	Specifically expressed in pre-B cell [50] (i)
FAM129C	NM_173544	B-cell novel protein 1	Specifically expressed in B-lymphocytes (k)
FCRL1	NM_052938	FC receptor-like 1	May serve as an activating coreceptor on B cells [51] (i)
FCRL2	NM_030764	FC receptor-like 2	Expression is limited to the mature B-cell lines (h); May have an regulatory role in normal and neoplastic B cell development (j)
FCRLA	NM_032738	FC receptor-like and mucin-like 1	Specifically expressed in B-cells(k)
NFE2L3	NM_004289	nuclear factor (erythroid-derived 2)-like 3	Highly expressed in B-cell (h)
MCOLN2	NM_153259	mucolipin 2	Might play a role in B cell lysosomal function [25] (i)
NT5E	NM_002526	5'-nucleotidase, ecto (CD73)	Mediates B cell adhesion [52] (i)
PAX5	NM_016734	paired box gene 5 (b-cell lineage specific activator)	Key regulator of the B-cell-restricted expression of the CD23a isoforms [53] (i)
TBK1	NM_013254	tank-binding kinase 1	TBK1-mediated signaling in haematopoietic cells was critical for the induction of antigen-specific B and CD4(+) T cells [54] (i)

Involvement in B cells was determined based on: (a) KEGG pathway; (b) GO Biological Process; (c) GO Cellular Component; (d) Biocarta; (e) BBID; (f) UniProt Tissue database; (g) UniProt Keywords database; (h) UniProt Tissue Specificity; (i) GENERIF_SUMMARY (PubMed); (j) UniProt Function; (k) GeneCards.

We further generated a combined list of genes included in all B cell-related annotations, using DAVID functional analysis tools (342 genes listed in Additional file 4, Table S3 from GO, KEGG, Biocarta, BBID, UniProt Tissue and UniProt Keywords databases; http://david.abcc.ncifcrf.gov/[23,24]). A significant enrichment of genes that carry B cell-related annotations was identified among the 115 changed genes (19/115, Tables 2A and 2B) compared to all genes in the core-level Exon 1.0 array (342/21,980, χ^2 ^test, χ^2 ^= 159.43, *P *< 0.0001), and to all genes expressed in our PBL samples (272/17,441, *P *< 0.0001, χ^2 ^test, χ^2 ^= 156.9, *P *< 0.0001). Furthermore, 11 additional genes that are associated with B cells were also detected via a manual search of the following databases: GeneCard, UniProt Tissue Specificity, UniProt Function and PubMed (Table 2C). In total, a high proportion (26%) of the significantly changed genes (30/115, Table 2) was related to B cell functions. Although some of these genes may carry additional roles, their common significantly enriched functions related to B cell biology. Notably, among these genes were *IGHM *and *IGHD *that are uniquely expressed in B cells, and the *CD19 *gene, which is frequently used as a marker for B cell identification.

### Positive correlations between the differentially expressed genes

**Figure 3:**
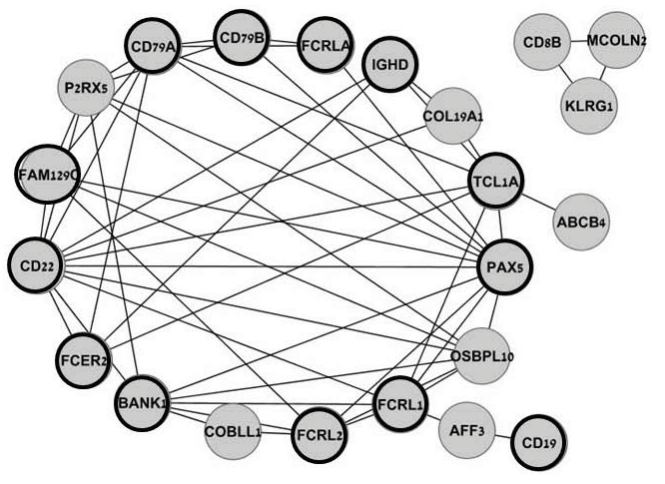
**B cell-related genes in the network of differentially expressed genes**. The Pearson Correlation test between the expression levels of the 115 differentially changed genes generated two gene networks. Highly significant correlations with a threshold of r > 0.8 and *P *< 10^-14 ^are presented here. B cell-related genes are in bold circles. Complete correlation data are available in Additional file 5, Table S4.

The Pearson Correlation test performed between the 115 significantly changed transcripts in all 59 patient and control samples identified 54 highly significant correlations (threshold: r > 0.8 and *P *< 10^-14^, Additional file 5, Table S4). Two gene-networks were generated based on these correlations (Cytoscape, Figure 3), suggesting a co-regulation of the gene expression. The large gene-network included multiple correlations between 19 genes; all were down-regulated in patients' PBL, most of them (16/19) were immune response genes (Additional file 4, Table S3). In particular, 13 of these 19 highly correlated genes are B cell-related (Table 2, bold circle in Figure 3). The second gene-network (Figure 3) linked 3 genes, *CD8B *and *KLRG1 *that are expressed in T cells, and *MCOLN2 *that was suggested to play a role in B cell lysosomal function [25].

### Validation

Of the 115 significantly changed genes, 10 genes were chosen for validation, representing both up- and down-regulated genes as well as different GO-annotations, including immune and defense responses (*CD22*, *CD19*, *CD79A, IGHD *and *IGHM*), regulation of cell proliferation and differentiation (*EREG*), regulation of transcription (*PAX5*), voltage-gated ion channel (*KCNH8*) and cell-cell adhesion (*PCDH9*). The *SNCA *gene, whose expression levels were not changed between patients and controls, was used as negative control. Validation of the expression results was done using RNA samples from 9 PD-patients and 6 controls that were also hybridized to the Human Exon 1.0 ST array. Highly significant positive correlations were demonstrated between the expression levels resulting from both methods in all tested genes [ranged between r = 0.864 (*P *< 0.0005) and r = 0.666 (*P *= 0.007); Additional file 6, Table S5].

### Are the expression levels of B cell-related genes affected by disease state, disease duration, medication or gender?

We expanded the number of RNA samples analyzed by Real-Time PCR (total of 79) to include an additional 64 samples from males and females patients and controls who were not tested on the microarrays. Six B cell-related genes were analyzed: *CD22*, *CD19 *and *CD79A *that encode B cell surface molecules, *IGHD *and *IGHM *that encode B cell specific immunoglobulins, and *PAX5 *that encodes a regulator of B cell genes. In order to define whether disease state (PD), rather than PD pharmacological therapy, affected the expression levels, we included 20 samples from patients who were not treated at the time of enrollment ("naïve"-PD group, with disease duration of 1.65 ± 1.42 years). Two additional groups of medically treated patients were included in this extended analysis: 18 patients with short disease duration (PD-SDD, less than 2 years between onset of motor symptoms to enrollment), and 20 with long disease duration (PD-LDD, more than 5 years between onset of motor symptoms to enrollment). Importantly, all four experimental groups of patients and controls included men and women in ~1:1 ratio (total of 39 men and 40 women).

**Figure 4:**
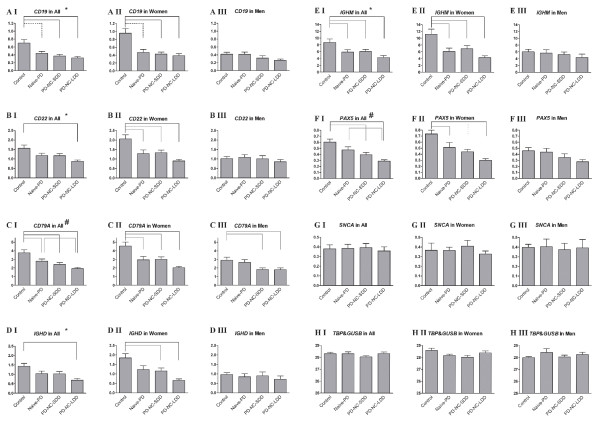
**Confirmation of the differential expression of B cell-related genes**. Quantitative Real-Time RT-PCR analyses were performed to confirm the expression changes in *CD19*, *CD22*, *CD79A*, *IGHD, IGHM *and *PAX5 *genes (**A-F**). *SNCA *gene expression was used as a negative control (**G**). Each transcript's expression level was normalized to the geometric mean of *GUSB *and *TBP *expression levels (**H**). Graphs in column I (AI-HI, Two-way ANOVA, followed by Tukey *post hoc*) represent the combined analysis of men and women (79 samples). Each analysis included a group of sex- and age-matched healthy controls, and three groups of patients: PD-patients that did not receive anti-Parkinson medications at time of enrollment (naïve-PD), PD-patients with short disease duration (PD-SDD) and PD-patients with long disease duration (PD-LDD). Each of these four groups included both women and men in a 1:1 ratio. Graphs in columns II and III (AII-HII and AIII-HIII, One-way ANOVA, followed by Tukey *post hoc*) represent the separate analysis of women (40 samples) or men (39 samples), respectively. For all gene analyses, bold numbers in AI-AIII represent the number of individuals tested. Bars represent mean ± SEM. Significant *P*-values are indicated between the tested groups as follows: black solid lines represent *P *< 0.0005, black dashed lines represent *P *< 0.005 and grey solid lines represent *P *< 0.05. Statistical significances were detected (Two-way ANOVA) for both gender and group parameters (#) and for gender and group parameters, as well as the interaction between them (*).

First, a 2-way ANOVA, with experimental group and gender as independent variables, revealed the significant effects of both parameters on the expression levels of all tested B cell-related genes (Figure 4AI-FI). Additionally, significant interactions between gender and experimental group were detected for *CD19*, *CD22*, *IGHD *and *IGHM *(Figure 4AI, BI, DI and 4EI). Next, men and women were analyzed separately. In men, none of the genes tested, except *CD79A *(Figure 4CIII), were significantly changed between the four experimental groups. In contrast, in women, all tested B cell-related genes were differentially expressed between the control group and the different PD-groups (1-way ANOVA, followed by Tukey *post hoc*, Figure 4AII-FII). Compared to controls, the expression of the 6 B cell-related genes was down-regulated in all female patient groups, naïve and treated (Figure 4AII-FII). Further decreased expression levels were noted for all tested genes, except for *CD19*, in the patient group with long disease duration (Figure 4B-F). Finally, the expression levels of the negative control gene, *SNCA*, and the two internal control genes, *TBP *and *GUSB*, were not changed between the four experimental groups, nor were they changed between men and women (Figures 4GI-III and 4HI-III). Taken together, these results demonstrate that the expression levels of these B cell-related genes are affected by the disease status, regardless of medication, mainly in women PD-patients.

### Studying the B cell related genes expression in other PD patient populations and in other neurological diseases

The expression levels of four of the B cell related genes (*IGHD*, *IGHM*, *CD19 *and *CD22*) were also examined in 10 non-Ashkenazi female PD patients and 11 non-Ashkenazi female controls. No changes in the expression levels of these genes were detected between PD-patients and controls (Additional file 7, Figure S2). Notably, the expression levels in non-Ashkenazi PD women (patients and controls) were similar to the expression levels in the Ashkenazi control women. These results might suggest that the observed changes in B cell related genes are specific to the Ashkenazi PD women population.

To examine the expression of the B cell related genes in other independent PD cohort and in patients with other neurological diseases we re-analyzed the previously published data by Scherzer *at al*. ([9]; GSE6613). This dataset compares PD patients to healthy controls and to other neurological disease controls. Based on our criteria for detecting differentially expressed genes (*P *< 0.01 and FC > 1.5 or < -1.5), no significant changes in the expression of the B cell related genes were observed between any of the three experimental groups, in the whole cohort or when genders were analyzed separately (with the exception of IL1R2, which was up-regulated in males PD patients and was also one of the only up-regulated B cell related genes in our data). These results might suggest that the changes we observed in the expression of B cell related genes are not common to other neurological diseases. However, since we could not confirm the changes also in the PD-patients it is possible that other factors might influenced these results, including demographic properties such as ethnicity and age of the female patients and controls, as well as technical aspects, such as the array used and the normalization method in the dataset of Scherzer *et al*.. For example, in the original article the authors used the MAS5 algorithm to normalize the array data, while in our re-analysis we utilized the GCRMA algorithm. As a result, out of the 24 probesets defined by Scehrzer *at al*. as differentially expressed (Supplementalry Table 3 [9]), only 8 probesets showed *P *< 0.01 when analyzing the data with GCRMA. Taken together, these results might suggest that the B cell related changes are specific to Ashkenazi Jewish PD female patients, however, the results could also be referred to other causes.

## Discussion

While transcriptional events in brain tissues from deceased PD patients have shed light on disease pathogenesis, recent studies have demonstrated the potential of PBL as a surrogate tissue in PD research. Mammalian lymphocytes are capable of synthesizing dopamine (DA) and other catecholamines, as well as DA receptors and transporters (DAT) [26,27]. Furthermore, when PBL from PD-patients were compared to controls, differences in DA signaling, including reduced DA content, impaired DAT immunoreactivity [28] and higher levels of dopamine D1-like and D2-like receptors, were observed [29]. The hypothesis that PBL can echo some of the changes occurring in the substantia nigra of PD-patients is also supported by our results. Changes in expression levels, in the same direction (up- or down-regulation), of the *ADCY2*, *CCDC92*, *CELSR1*, *HECTD2 *and *KIF1B *genes, were demonstrated both here, in patients' PBL, and previously, in the substantia nigra of PD-patients [6,19]. In addition, genes involved in PD-related pathways, such as the ubiquitin-proteasome and apoptosis, and mitochondrial function were differentially expressed in patients' PBL both in our study and in recent reports [9,10]. These results suggest that PBL from PD-patients serve as an important, easily accessible tool, that might help in the study of mechanisms underlying Parkinson's disease pathogenesis. Our data also demonstrated that the selection of a relatively homogeneous group of RNA samples from Ashkenazi women that do not carry either *GBA *or *LRRK2 *founder mutations [14,15] increased the ability to detect novel expression changes in PD patients' PBL.

The involvement of the immune system, and particularly T cells, in PD has been recognized (reviewed by [30]). Our expression analysis of PBL from PD-patients demonstrated that the most prominent group of differentially expressed genes were those involved in immune system processes. Notably, a significantly decreased expression of innate and humeral immune response genes was detected, mainly of genes related to B cell functions. The down-regulation of dozens of genes encoding the CD surface molecules, the B cell specific immunoglobulins IGHM and IGHD, and regulators of B cell differentiation and activation, suggest a decrease in the B cell population among women with PD. Such a decrease in the number and percentage of peripheral CD19^+ ^B cells was previously demonstrated in PD-patients [31]. Indeed, decreased mRNA levels of *CD19 *were confirmed in our study by both microarray and quantitative real-time PCR analyses. Changes in peripheral B and T lymphocytes were also described in other neurodegenerative disorders, such as Alzheimer's disease [32] and amyotrophic lateral sclerosis [33]. Our findings suggest that B cells might in fact be an additional tissue involved in PD, which has been recently appreciated as multi-systemic, beyond the central nervous system, involving the enteric and autonomic nervous systems as well as the eye (reviewed by [34] and [35]).

The detection of expression changes in tissues taken from patients under pharmacotherapy raises the question of whether changes are related to the disease, therapy, or both. Therefore, the quantitative expression confirmation analysis included both treated and untreated (naïve) patients. Our data demonstrated that the reduced expression levels of B cell-related genes did not result from anti-parkinsonian medications, but were related to the presence of PD. This is in agreement with the decreased number and percentage of CD19^+ ^B cells observed in both treated and untreated PD-patients [31].

While our initial microarray expression study tested only women, we greatly expanded the number of samples for the confirmation analysis, testing six B cell-related genes in male and female PD-patients and controls. Interestingly, the expression levels of all six genes were significantly under-expressed only in women patients compared to women controls, whereas in men, the expression levels were similarly lower in both patients and controls. Such a gender-dependent differential expression of B cell-related genes is reminiscent of the variations detected in the levels of different subsets of lymphocyte populations between healthy men and women [36,37], specifically, the higher count of CD19^+ ^B cells in healthy women compared to men [38]. Additionally, since our analysis of Ashkenazi PD women samples demonstrated an expression changes in B cell related genes both in the initial stage and in the confirmation study, and given that we could not detect the same changes in expression patterns in non-Ashkenazi PD women (in Jewish women with PD from Eastern and North-African origin, as well as in Scherzer *et al*. [9] cohort), it is possible that these changes might be specific to Ashkenazi PD women.

Gender-associated phenotypes are well recognized in PD, particularly the greater prevalence and the younger age of motor symptom onset in men [17]. Specific differences were also detected in peripheral blood, where serum uric acid levels have been inversely correlated with disease duration and daily levodopa dosage in male, but not female PD patients [39]. Recently, gender-dependent gene expression changes were demonstrated in the SN of PD-patients, with only a small overlap of the differentially expressed genes between males and females [40]. The expression changes detected in B cell-related genes in our study are yet another example of molecular variations between men and women with PD. It will be important to explore the relationship between the down regulation of B cell-related genes and the development of PD: Are women with down regulation of B cell-related genes more prone to develop PD, with a higher expression of B cell-related genes possibly carrying a protective effect? or is it the disease process itself that decreases the expression of B cell-related genes in women?

## Conclusions

The results presented here demonstrated down-regulated expression of large number of B cell related genes, including genes that are specifically expressed in B cells and genes encoding B cell surface molecules. Moreover, we showed that their expression decreased specifically in women PD-patients. Our results provide compelling evidence for the involvement of B cells in PD even before the initiation of anti-Parkinsonian drugs. Further studies will be needed to elucidate the possible roles of the B cell down-regulation in PD pathogenesis.

## Methods

### Patients and controls

All PD-patients and controls tested here were included in our previous studies [14,15,41], and the diagnostic criteria, modes of recruitment and genotyping of *LRRK2 *and *GBA *mutations were described therein. In the first step of our study, the microarray analysis, we included RNA samples from female patients and controls who were not carriers of either the *LRRK2 *G2019 or *GBA *mutations. A random selection of 30 patients (age at enrollment, i.e. age at blood sampling 65.7 ± 10.3, range 47-84; age at motor symptoms onset 56 ± 11.58, range 26-84) was done using SPSS software Version 16 (SPSS Inc., Chicago, IL) from a pool of 86 non-carrier female patients. The 29 controls were randomly selected to match the mean, SD and range of the age at enrollment of the patients, from a group of 91 healthy females (age at enrollment of 65.48 ± 8.89, range 48-82). For the second step of the study, the quantitative RT-PCR (q-RT-PCR) analysis, we tested 79 RNA samples from males and females (1:1.03 ratio), PD-patients and controls (58 and 21, respectively). Twenty patients were naïve, untreated, and 38 were under PD pharmacotherapy. None were carriers of either the *LRRK2 *G2019S or *GBA *mutations. Fifteen of the 59 samples that were analyzed by the microarrays in the first step were randomly selected for validation by q-RT-PCR. In total, 123 RNA samples were included in these studies (84 females and 39 males). Table 3 summarizes the age and gender of patients and controls that were used for the q-RT-PCR analysis. All study samples were from individuals of un-related Ashkenazi-Jewish ancestry. q-RT-PCR was also performed using 10 non-Ashkenazi Jewish female PD patients (age at enrollment of 61.7 ± 8.4) and 11 age- and ethnicity-matched female controls. Each group included females from North Africa and Eastern (Iraq, Iran, Syria) origin. All patients and controls signed an informed consent and the study was approved by the Institutional and National Supreme Helsinki Committees for Genetic Studies.

**Table 3:** Age at enrollment (AAE) and age at onset (AAO) of the patients' and controls' samples used for the q-RT-PCR assays

GROUP	Sex	No	AAE	AAO
Control	F	11	61.45 ± 9.10	
Control	M	10	64.30 ± 8.07	
Naive-PD	F	10	62.30 ± 12.82	60.50 ± 12.79
Naive-PD	M	10	64.50 ± 9.38	62.90 ± 10.08
PD-LDD	F	10	65.70 ± 7.82	51.80 ± 10.79
PD-LDD	M	10	67.10 ± 10.58	57.50 ± 12.83
PD-SDD	F	9	63.00 ± 8.14	62.11 ± 8.37
PD-SDD	M	9	64.89 ± 8.65	64.44 ± 8.85

None of the groups showed significant difference in AAE.

No, Number of samples used.

### RNA isolation, target labeling and hybridization

PBL were obtained from 2.5 mL of peripheral blood that was lyzed within 2 h from blood taking (buffer EL, QIAGEN, Germantown, MD). The PBL pellets were immediately frozen and kept at -80°C. Total RNA was extracted using QIAamp RNA blood Mini Kit in the presence of DNase (QIAGEN). RNA quality and quantity were measured using NanoDrop spectrophotometer ND-1000 (NanoDrop Technologies, Wilmington, DE). Only RNAs that passed the quality control cutoffs of 260/280 ratio of 1.8 and 260/230 ratio of 1.6 were used. GeneChip whole-transcript sense target labeling of 1 μg of total RNA was done according to the manufacturer's instructions (GeneChip WT cDNA Synthesis and Amplification and GeneChip WT Terminal Labeling Kits, Affymetrix Inc., Santa Clara, CA). The labeled targets were hybridized to the Affymetrix GeneChip Human Exon 1.0 ST Arrays. Hybridized arrays were washed and stained on a GeneChip Fluidics Station 450 and scanned on a GCS3000 Scanner (Affymetrix). Target labeling, hybridization and scanning were done in batches of 8 samples, each containing an equal number of samples from all experimental groups. Microarray experiments were designed to comply with minimum information about a microarray experiment (MIAME) guidelines [42].

### Statistical and bioinformatic analyses

Normalization, filtering and statistical analyses were done using Partek^® ^Genomics Suite Version 6.4 (Partek Inc., St. Louis, MO). Background adjustment, normalization, and probe-level summarization of the microarray data were done using the robust multichip average (RMA) algorithm [43] and summarized signals were log2 transformed. Gene-level summarization was done by calculating the mean signal for each gene, based on the meta-probeset file provided by Affymetrix. To eliminate the batch effects (scan date and personnel), the ANOVA-batch-removal tool implanted in Partek was applied. The first filtering step was done to include only the core-level data of the Human Exon 1.0 ST array as defined by Affymentrix, which includes 21,980 gene and 232,448 exon probesets, consisting of RefSeq and full-length GenBank mRNAs. To avoid analysis of non-expressed genes, only exons with signal values of ≥ 3.0 were selected for further analysis. To detect transcriptional changes, 3-way ANOVA was conducted on the exon-level data, with the disease status and the two batch-removed effects as the ANOVA factors. To eliminate false-positive results due to probes that site on SNPs [44], differentially expressed exon-level probesets that contained SNPs in more than 50% of their probes were identified using the SNPinProbe1.0 database [45] and removed. *P*-values for the ANOVA model were calculated using log-transformed data. Expression fold changes were calculated by least squares (LS) mean. Since Partek's alternative-splicing ANOVA algorithm is affected by exon-to-exon differences, Partek's gene view tool was used to determine alternatively-spliced genes.

Pearson correlation test was used to examine possible correlations between expression levels of the differentially expressed transcripts, which were employed for expression-based network (Expression correlation plug-in at Cytoscape software 2.6.1 [46]). To determine functional significance among the changed transcripts, over-represented gene ontology (GO) and KEGG pathways were identified, compared to their representation on the Human Exon 1.0 ST array, using the online software WebGestalt (http://bioinfo.vanderbilt.edu/webgestalt/[47]).

### Quantitative RT-PCR analysis

cDNA was synthesized from 0.5 μg total RNA using High-Capacity cDNA Reverse Transcription Kit (Applied Biosystems, Foster City, CA) in the presence of an RNase inhibitor. The expression levels of the following genes were validated: *CD19 *(Hs01047409_g1), *CD22 *(Hs00233533_m1), *CD79A *(Hs00998120_g1), *EREG *(Hs00914313_m1), *KCNH8 *(Hs00976951_m1), *IGHD *(Hs00378878_m1), *IGHM *(Hs00378435_m1), *PAX5 *(Hs01045956_m1) and *PCDH9 *(Hs01009199_m1). The expression of *SNCA *(Hs01103386_m1) was used here as negative control, since its expression levels were not changed between the studied groups in the Affymetrix Exon 1.0 array experiment (data not shown). The expression levels of all tested genes were normalized relative to the expression levels of the *GUSB *(Hs99999908_m1) and *TBP *(Hs99999910_m1) genes. These reference genes were selected based on their microarray expression out of several candidate reference genes using NormFinder algorithm, which allows the selection of genes with the least expression changes between the different samples and the experimental groups ([48], data not shown). Quantification was done using TaqMan MGB probe and TaqMan Universal PCR Master Mix with 10 ng of cDNA on the StepOne Plus Real-Time PCR system (Applied Biosystems). Reactions were performed for 2 min at 50°C, 10 min at 95°C, and then 45 cycles of 15 s at 95°C and 1 min at 60°C. The expression levels were determined using the comparative threshold cycle (C_T_) quantification method [49]. The geometric mean of C_T _values of the internal control genes, *GUSB *and *TBP*, was subtracted from C_T _values of each target genes (ΔC_T_) and signal values are expressed as 2 ^(-ΔCT)^. Pearson correlation test was applied to examine the correlation between results obtained from the Exon 1.0 ST Array and the Real-Time PCR. The expanded confirmation analysis (in 79 samples from men and women) included the following genes: *CD19*, *CD22*, *CD79A*, *IGHD*, *IGHM*, *PAX5 *and *SNCA*. Their expression signal values were compared between the study groups, and 2-way ANOVA with gender and group as the two independent variables, followed by 1-way ANOVA statistics were applied.

### Analyzing published blood expression profile data of Parkinson's and other neurological diseases

To detect whether the expression changes in B cell related genes seen in our PD patients can be also detected in other neurological diseases' patients and in other PD cohort, we re-analyzed the published data of Scherzer *et al*. ([9]; GSE6613). This dataset includes 50 PD patients, 22 healthy controls and 33 controls with other neurological diseases. The CEL files were downloaded to Partek^® ^Genomics Suite Version 6.5. Background adjustment and normalization of the microarray data were done using the GCRMA algorithm. One sample of healthy control (GSM155358) was detected as an outlier by PCA, and was removed from further analyses. Since the gender of the subjects was not provided, we used the expression of *XIST *to distinguish between males and females. According to the *XIST *expression, the cohort included 41 females (12 PD patients, 11 healthy controls and 18 other neurological diseases controls) and 63 males (38 PD patients, 11 healthy controls and 15 other neurological diseases controls). ANOVAs were done to detect significant changes between PD patients, healthy controls and controls with other neurological diseases among: all subjects, only females and only males.

## Competing interests

The authors declare that they have no competing interests.

## Authors' contributions

AOU conceived, designed and supervised the study. MK and ABS performed the molecular analyses. MK analyzed the data. NG and TG recruited the patients. MK and AOU wrote the manuscript. NG, ABS and TG reviewed the manuscript. All authors read and approved the final manuscript

## Supplementary Material

Additional file 1 **Supplemental Figure 1: PCA mapping based on global gene expression**. PCA mapping of all RNA samples based on the expression levels of all exon probesets demonstrated a separation of the samples to groups according to two methodological factors: (**A**) Two different kit's batches (Affymetrix whole-transcript target labeling kit) that were reflected by the scan date, and (**B**) Two different researchers that extracted the RNAs. (**C**) and (**D**), the PCA mapping that was done after the removal of these two methodological effects, respectively, demonstrated homogeneity of data. Click here for file

Additional file 2 **Supplemental Table 1: Differentially expressed genes**. List of the 115 genes (and 206 probesets) whose expression levels were significantly changed between PD-patients and controls. Asterisks (*) denote alternatively-spliced genes. Click here for file

Additional file 3 **Supplemental Table 2: Enriched GO-annotations in the 115 differentially expressed genes**. Full list of the significantly over-represented GO-annotations among the 115 significantly changed genes. O, Observed number of genes carrying the annotation among the 115 differentially expressed genes; E, expected number of genes that carry this annotation among all core-level genes on the array; R, ratio between observed and expected; P, *P*-value of the enrichment, calculated using Fisher exact test. Click here for file

Additional file 4 **Supplemental Table 3: List of B cell-related genes on the Exon 1.0 array**. Combined list of all B cell-related genes that are represented on the core-level Exon 1.0 array, which were extracted based on their annotations from multiple databases (DAVID functional analysis tools: GO, KEGG, Biocarta, BBID, UniProt Tissue and UniProt Keywords databases [23,24]). Click here for file

Additional file 5 **Supplemental Table 4: Summary of Pearson correlation analysis**. A list of all correlated genes detected among the 115 significantly changed transcripts within all 59 RNA samples (Pearson Correlation test, threshold: r > 0.8 and *P *< 10^-14^). Click here for file

Additional file 6 **Supplemental Table 5: Validation of expression analysis**. Results of the validation analyses that tested the expression levels of 10 genes in 9 PD-patient and 6 control samples. Detailed are the mean and SD obtained by both the Human Exon 1.0 ST array and the quantitative real-time RT-PCR analyses, as well as the results and significance of the Pearson Correlation test, which compared the expression levels that resulted from these two methods. Click here for file

Additional file 7 **Supplemental Figure 2: B cell-related genes expression in non-Ashekanazi PD patients**. Quantitative Real-Time RT-PCR analyses were performed to confirm the expression changes in *IGHD*, *IGHM*, *CD19 *and *CD22 *in 10 non-Ashkenazi female PD patients and 11 non-Ashkenazi female controls. Each transcript's expression level was normalized to the geometric mean of *GUSB *and *TBP *expression levels. Bars represent mean ± SD. Click here for file
