# Supplementary figures and images for: Decreased expression of B cell related genes in leukocytes of women with Parkinson's disease
Source: Mol Neurodegener. 2011 Sep 23;6:66. doi: 10.1186/1750-1326-6-66 (PMC3189133; doi:10.1186/1750-1326-6-66)

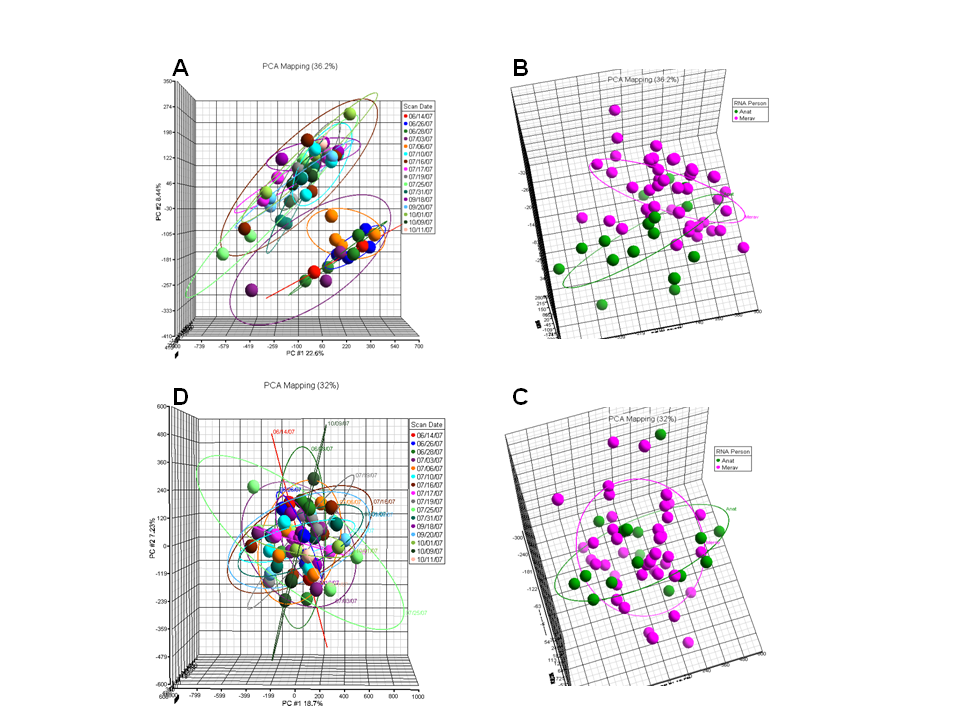

Supplement: Additional file 1 — Supplemental Figure 1: PCA mapping based on global gene expression. PCA mapping of all RNA samples based on the expression levels of all exon probesets demonstrated a separation of the samples to groups according to two methodological factors: (A) Two different kit's batches (Affymetrix whole-transcript target labeling kit) that were reflected by the scan date, and (B) Two different researchers that extracted the RNAs. (C) and (D), the PCA mapping that was done after the removal of these two methodological effects, respectively, demonstrated homogeneity of data. [file 1750-1326-6-66-S1.TIFF]

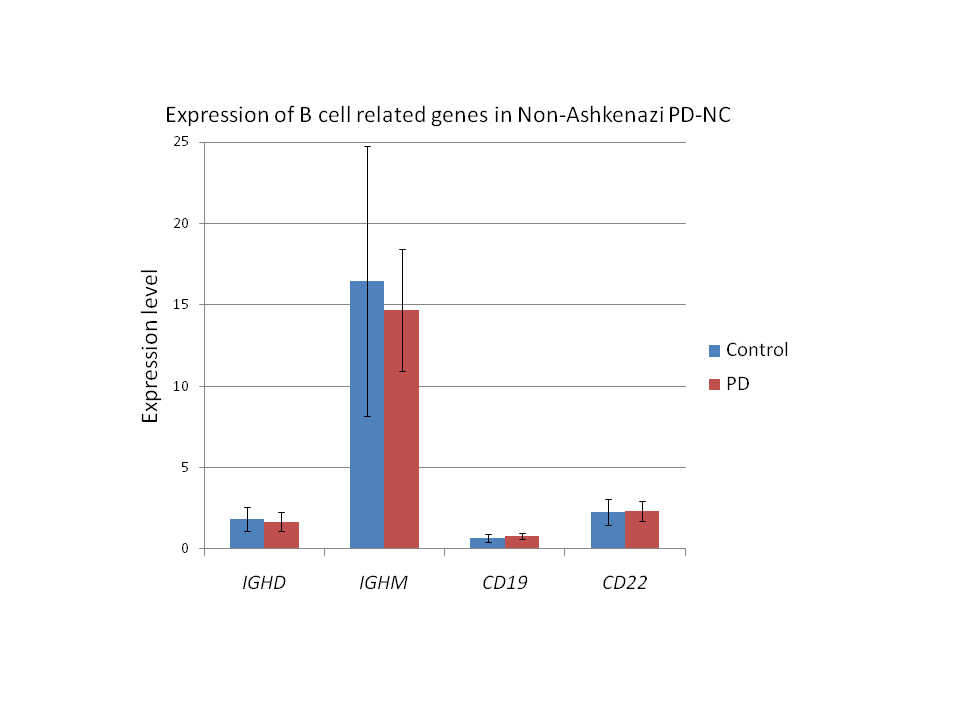

Supplement: Additional file 7 — Supplemental Figure 2: B cell-related genes expression in non-Ashekanazi PD patients. Quantitative Real-Time RT-PCR analyses were performed to confirm the expression changes in IGHD, IGHM, CD19 and CD22 in 10 non-Ashkenazi female PD patients and 11 non-Ashkenazi female controls. Each transcript's expression level was normalized to the geometric mean of GUSB and TBP expression levels. Bars represent mean ± SD. [file 1750-1326-6-66-S7.TIFF]
